# Rapidly Progressive Glomerulonephritis Associated With Anti-glomerular Basement Membrane Disease: A Rare Diagnosis

**DOI:** 10.7759/cureus.76469

**Published:** 2024-12-27

**Authors:** Catarina Maia, João Rocha, Henrique Cardoso, Teresa Antunes, Jorge S Almeida

**Affiliations:** 1 Oncology, Centro Hospitalar Universitário de São João, Porto, PRT; 2 Internal Medicine, Centro Hospitalar Universitário de São João, Porto, PRT; 3 Physical Medicine and Rehabilitation, Centro Hospitalar Universitário de São João, Porto, PRT; 4 Medicine, Faculdade de Medicina da Universidade do Porto (FMUP), Porto, PRT

**Keywords:** anti-glomerular basement membrane disease, case report, glomerulonephritis, goodpasture syndrome, rapidly progressive glomerulonephritis

## Abstract

Anti-glomerular basement membrane disease is a rare small vessel vasculitis caused by the deposition of immunoglobulin G (IgG) autoantibodies in the basement membrane of glomerular capillaries and lung alveoli, leading to rapidly progressive renal failure and/or alveolar hemorrhage. We report the case of an 83-year-old female patient presenting with uremic symptoms, rapidly progressive kidney failure, and a high titer of anti-glomerular basement membrane antibodies. Given the urgent need for kidney replacement therapy, the substantial fibrosis and glomerular scarring observed in the kidney biopsy suggesting a chronic process, and the absence of pulmonary involvement, neither immunosuppressive treatment nor plasmapheresis was initiated, since a low likelihood of a favorable response to these interventions was expected. The patient started intermittent hemodialysis, which resulted in significant clinical improvement. This case highlights the importance of considering anti-glomerular basement membrane disease in the differential diagnosis of rapidly progressive kidney failure, as early diagnosis and timely treatment may be crucial for reducing the risk of progression to end-stage kidney disease.

## Introduction

Anti-glomerular basement membrane (anti-GBM) disease is a small-vessel vasculitis caused by immunoglobulin G (IgG) autoantibodies against the non-collagenous domain (NC1) of the α3 chain of type IV collagen, present in the basement membrane of glomerular capillaries and pulmonary alveoli. Deposition of these autoantibodies and the associated immune-mediated injury may result in crescentic glomerulonephritis with rapidly progressive renal failure (RPRF) and alveolar hemorrhage with pulmonary-renal syndrome (Goodpasture's syndrome). Presentation with RPRF occurs in up to 90% of cases while alveolar hemorrhage may occur in 40-60% of cases, mostly in men during the second decade of life [1]. Anti-GBM disease is rare, with an estimated incidence of 0.5-1 cases per million people annually [2], so clinical suspicion is important for diagnosis. Early diagnosis and prompt intervention with immunosuppression and plasma exchange are crucial for preventing progression to end-stage kidney failure, alveolar hemorrhage, and death [3]. We report a case of a patient with anti-GBM disease presenting with RPRF and crescentic glomerulonephritis, highlighting the importance of considering this rare disease in the differential diagnosis.

## Case presentation

An 83-year-old female patient was admitted to the emergency department with complaints of decreased urine output and black stools over the past three days, accompanied by asthenia, anorexia, weight loss, bilateral ankle edema, and bilateral feet and calf cramps lasting for one month. No complaints of fever, abdominal pain, vomiting, diarrhea, dyspnea, cough, hemoptysis, hematuria, or foamy urine were reported. There was no history of recent infections or introduction of new medication, including non-steroidal anti-inflammatory drugs. Her medical history included essential arterial hypertension, treated with perindopril 8 mg daily, and chronic kidney disease (stage 3aA2 according to the KDIGO (Kidney Disease: Improving Global Outcomes) classification) [4], with a serum creatinine level of 1.00-1.15 mg/dL evaluated eight months before admission.

On physical examination, the patient presented with arterial hypertension (163/89 mmHg), bilateral ankle edema, and jugular venous distension observed in the 30º recumbent position. No periorbital puffiness was observed. Cardiac and pulmonary auscultation were unremarkable. Abdominal examination revealed no abnormalities, and melena was identified during digital rectal examination.

**Figure 1:**
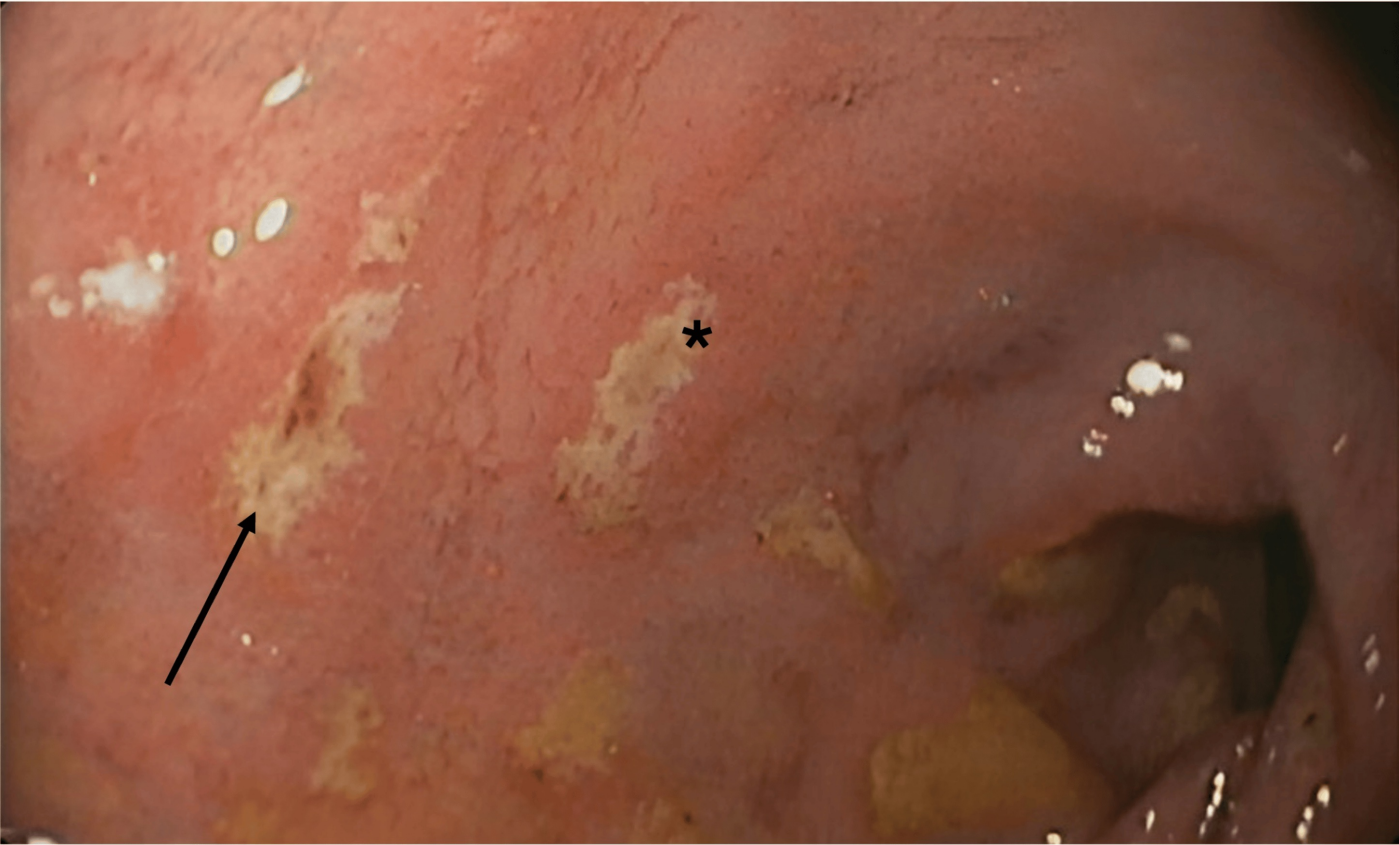
Upper endoscopy findings Duodenal inspection revealed several ulcers, classified as Forrest IIc (arrow) and Forrest III (*).

Laboratory tests revealed anemia (Hb 6.6 g/dL), severe impairment of renal function (creatinine 24.8 mg/dL, urea 483 mg/dL), and hyperkalemia (8.7 mEq/L) (Table 1). A summary urine test showed leukocyturia and erythrocyturia, with an albumin-creatinine ratio of 4124 mg/g of creatinine, and no evidence of erythrocyte casts or dysmorphic erythrocytes. Severe metabolic acidosis was found in arterial blood gas analysis. Renal ultrasound demonstrated normal-sized kidneys without hydronephrosis, and chest radiography revealed no abnormal findings. Due to the presence of anemia and melena, an urgent upper digestive endoscopy was performed, revealing Forrest IIc and III duodenal ulcers [5], as can be seen in Figure 1. Due to the severity of uremia and metabolic disturbances, urgent renal replacement therapy (RRT) was started with hemodialysis. The patient also received two units of red blood cells, with no transfusion reactions observed, and was started on a high-dose proton pump inhibitor.

**Table 1:** Relevant laboratory tests performed

Serum biochemistry parameters	Values presented	Normal values
Hemoglobin (g/dL)	6.6	12.0 - 16.0
White blood cell count (/μL)	8970	4000 - 11000
Platelets (/μL)	328000	150000 - 400000
Erythrocyte sedimentation rate (mm/1st h)	100	0 - 30
Albumin (g/L)	26.4	38.0 - 51.0
Urea (mg/dL)	483	10 - 50
Creatinine (mg/dL)	24.8	0.5 - 0.9
Sodium (mEq/L)	133	135 - 145
Potassium (mEq/L)	8.7	3.5 – 5.3
Chloride (mEq/L)	104	95-110
C-reactive protein (mg/L)	82	< 3.0
Arterial blood gas analysis		
pH	7.13	7.35 - 7.45
Partial pressure of O2 (mmHg)	119	80 – 100
Partial pressure of CO2 (mmHg)	17	35 – 45
Bicarbonate (mEq/L)	5.7	22 – 26
Lactate (mmol/L)	0.5	< 2.0
Urinary biochemistry parameters		
White blood cell count (/μL)	13427	< 30
Urinary RBC (/uL)	1676	< 27
Albumin/creatinine ratio (mg/g of creatinine)	4124	< 30

**Figure 2:**
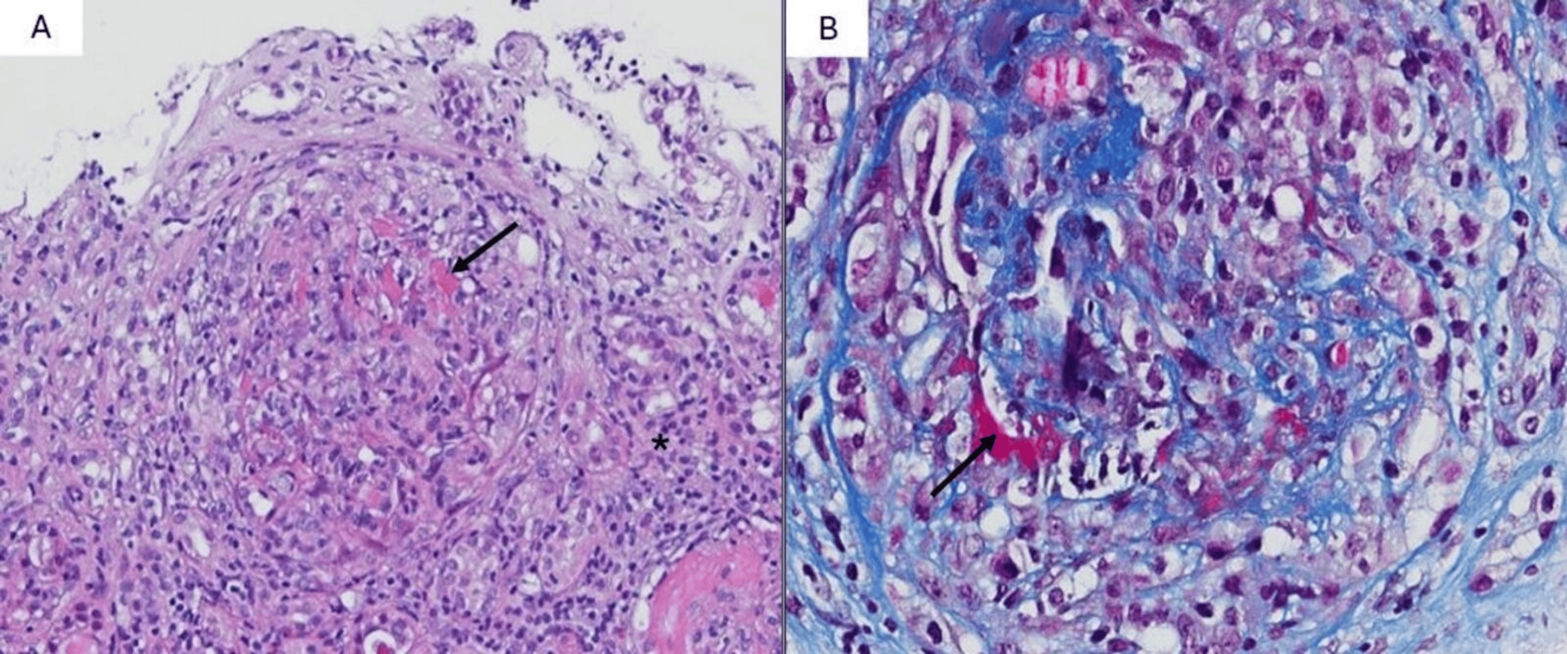
Anatomopathological findings of renal biopsy A total of 21 glomeruli were identified in the renal biopsy: four sclerosed glomeruli, one preserved glomerulus, and the remaining glomeruli exhibited cellular crescents with fibrinoid necrosis (arrows), as evidenced in panels A (hematoxylin and eosin stain) and B (Masson's trichrome stain). The renal interstitium displayed a polymorphic inflammatory infiltrate, predominantly composed of lymphocytes (*), with occasional eosinophils and plasma cells. Significant interstitial fibrosis and marked tubular atrophy were noted, affecting more than 60% of the represented parenchyma.

The patient was admitted to the Internal Medicine ward for further evaluation. A suspicion of RPRF was raised, attending to the uremic symptoms with a one-month evolution, the rapid and severe deterioration of kidney function, the absence of apparent pre- or post-renal causes, and the presence of significant leukoerythrocyturia and albuminuria. As described in Table 2, the immunological study revealed elevated titers of anti-GBM antibodies (333 U/mL) and antinuclear antibodies (ANA, 1/640, mottled pattern), with positive anti-SSA and anti-Ro52 antibodies. A renal biopsy was performed, which demonstrated crescentic glomerulonephritis, interstitial fibrosis, and marked tubular atrophy affecting more than 60% of the observed parenchyma (Figure 2). The direct immunofluorescence study showed linear IgG deposition in the glomerular basement membrane, confirming the diagnosis of anti-GBM disease.

**Table 2:** Immunological study performed ANA: Antinuclear antibodies. ANCA: Anti-neutrophil cytoplasmic antibodies. dsDNA: double-stranded DNA. ENA: Extractable nuclear antigen. GBM: glomerular basement membrane. ELISA: Enzyme-linked immunosorbent assay.

Immunological tests	Values presented	Normal values
C3c (mg/dL)	100	83 - 177
C4 (mg/dL)	27	12 - 36
Immunoglobulin G (mg/dL)	1100	600 – 1560
Immunoglobulin A (mg/dL)	236	50 – 373
Immunoglobulin M (mg/dL)	25	40 – 325
ANA (titre)	1/640, speckled pattern	< 1/160
Antibodies anti-dsDNA	< 10	< 100
Antibody anti-GBM (UI/mL)	333	<7.0
Rheumatoid factor (UI/mL)	40.3	< 30
ENA panel (ELISA)	SS-A +++ Ro52 +++	N/A
ANCA (U/mL)	< 2	< 2

After starting RRT, the patient showed significant clinical improvement, with the resolution of her complaints. However, no recovery of renal function was observed, so a maintenance hemodialysis regimen was initiated. Considering the urgent need for RRT, the significant fibrosis documented, the lack of renal function recovery, and the absence of pulmonary involvement, it was decided not to initiate immunosuppressive therapy or plasmapheresis. During hospitalization, no recurrence of gastrointestinal bleeding was observed. High-dose proton pump inhibitor therapy was maintained, and the diet was gradually reintroduced with good tolerance. The patient was discharged after 20 days and continued her medical follow-up at the primary healthcare level.

## Discussion

This case report presents a typical presentation of anti-GBM disease manifesting as RPRF. Although rare, anti-GBM disease should be considered in the differential diagnosis of RPRF because early treatment is essential to prevent progression to end-stage renal disease, severe complications such as alveolar hemorrhage, and death [3]. As this case illustrates, anti-GBM disease should not be considered only in patients with lung-kidney syndrome since up to 40% of patients may not have pulmonary involvement [1]. Isolated renal involvement most commonly occurs in the sixth and seventh decades of life [1].

The diagnosis of anti-GBM disease includes serological testing and renal biopsy. Initial serological evaluation should measure anti-GBM antibodies, as well as other autoantibodies associated with other diseases in the differential diagnosis for RPRF such as ANA, anti-extractable nuclear antigens (ENA), and anti-neutrophil cytoplasmic antibodies (ANCA) [2]. Up to one-third of patients with anti-GBM disease may also have ANCA positivity [6]. Identifying these cases is important, attending to the higher risk of recurrence and the need for maintenance therapy, in contrast to anti-GBM disease without ANCA antibodies, where recurrence is rare [2,7]. Despite the fundamental role of anti-GBM antibodies in the diagnosis, they may be undetectable in up to 10% of cases, emphasizing the critical role of renal biopsy [2,8].

A renal biopsy should be performed ideally within the first 24 hours of presentation [2]. The presence of crescentic glomerulonephritis and linear deposition of IgG along the glomerular basement membrane on immunofluorescence are typical features of anti-GBM disease and may be important for differential diagnosis, especially in the presence of other autoantibodies, as demonstrated in this case [1]. In anti-GBM disease, the extent of glomerular damage is strongly correlated with the degree of renal dysfunction [9]. Oligoanuria and the urgent need for RRT at presentation are also associated with poor prognosis [10]. In patients requiring urgent RRT, it is estimated that only 8% recover renal function after one year [1]. Therefore, considering the risks associated with immunosuppression and the presence of the poor prognostic factors described, a conservative approach may be considered, especially when the renal biopsy shows more than 85% of glomeruli involvement [2]. In this case, due to the severity of renal function impairment and the need for urgent RRT, immunosuppressive therapy was not initiated, given the low probability of renal recovery. This decision was further supported by the histological findings of the renal biopsy, which revealed 95% of glomeruli affected and extensive interstitial fibrosis. Despite the lack of benefit in renal function recovery, starting treatment with immunosuppression and plasmapheresis would have been necessary if alveolar hemorrhage had been found, which was promptly ruled out [2].

## Conclusions

Anti-GBM disease is a rare yet potentially severe condition that requires early recognition and timely intervention to prevent progression to end-stage renal failure or severe complications such as alveolar hemorrhage. As demonstrated in this case, rapid deterioration of renal function, without pulmonary involvement, can be seen in isolated renal anti-GBM disease, especially in elderly patients. A high clinical suspicion, along with serological testing and renal biopsy, are crucial for a definitive diagnosis.

Given the high risk of irreversible renal damage, early intervention with immunosuppressive therapy and plasmapheresis is typically recommended. However, in cases with an urgent need for RRT and significant renal fibrosis, a conservative approach may be justified, as the likelihood of renal recovery is low. This case highlights the importance of individualized treatment decisions based on the patient's clinical condition, renal biopsy results, and the presence of prognostic factors such as the extent of glomerular involvement. Early diagnosis and management remain key factors in improving outcomes for patients with anti-GBM disease.
